# Facilitating the ethical use of health data for the benefit of society: electronic health records, consent and the duty of easy rescue

**DOI:** 10.1098/rsta.2016.0130

**Published:** 2016-12-28

**Authors:** Sebastian Porsdam Mann, Julian Savulescu, Barbara J. Sahakian

**Affiliations:** 1 Department of Psychiatry, Addenbrooke's Hospital, University of Cambridge, Cambridge CB2 0QQ, UK; 2 Center for Bioethics, Harvard Medical School, Boston, MA 02115, USA; 3 Oxford Uehiro Centre for Practical Ethics, Suite 8, Littlegate House, St Ebbes Street, Oxford OX1 1PT, UK; 4 Behavioral and Clinical Neuroscience Institute, Herchel Smith Building for Brain and Mind Sciences, Forvie Site, Robinson Way, Cambridge CB2 0SZ, UK

**Keywords:** informed consent, electronic health records, privacy, duty of easy rescue, selection bias, research ethics

## Abstract

Advances in data science allow for sophisticated analysis of increasingly large datasets. In the medical context, large volumes of data collected for healthcare purposes are contained in electronic health records (EHRs). The real-life character and sheer amount of data contained in them make EHRs an attractive resource for public health and biomedical research. However, medical records contain sensitive information that could be misused by third parties. Medical confidentiality and respect for patients' privacy and autonomy protect patient data, barring access to health records unless consent is given by the data subject. This creates a situation in which much of the beneficial records-based research is prevented from being used or is seriously undermined, because the refusal of consent by some patients introduces a systematic deviation, known as selection bias, from a representative sample of the general population, thus distorting research findings. Although research exemptions for the requirement of informed consent exist, they are rarely used in practice due to concerns over liability and a general culture of caution. In this paper, we argue that the problem of research access to sensitive data can be understood as a tension between the medical duties of confidentiality and beneficence. We attempt to show that the requirement of informed consent is not appropriate for all kinds of records-based research by distinguishing studies involving minimal risk from those that feature moderate or greater risks. We argue that the duty of easy rescue—the principle that persons should benefit others when this can be done at no or minimal risk to themselves—grounds the removal of consent requirements for minimally risky records-based research. Drawing on this discussion, we propose a risk-adapted framework for the facilitation of ethical uses of health data for the benefit of society.

This article is part of the themed issue ‘The ethical impact of data science’.

## 1. Introduction

The digitization of medicine presents healthcare professionals with novel opportunities for analysing medical information. Electronic health records (EHRs) and other electronic repositories of patient information offer several advantages over traditional paper records. Systematic reviews have found that electronic records are associated with increased efficiency and quality of care, thus freeing up the resources of health professionals [1–4].

Electronic records are easily manipulated, aggregated and shared, allowing cases to be discussed and expert opinions to be sought and provided globally. Whereas data in paper records have to be laboriously identified and abstracted by hand, EHRs can be interrogated to identify relevant data which can be copied and compiled electronically. These records can be combined to create much larger datasets than would be feasible manually, facilitating biomedical, epidemiological and public health research [5,6]. Derived from real clinical encounters, EHR data can be used to answer research questions that would be unethical to pursue in interventional research, such as the effects of exposure to toxins, clinical error and varying levels of access to health services [7]. EHR data are generated for all patients, and thus could be used to address the underrepresentation of minorities and vulnerable groups in interventional research [7]. Monitoring of records could lead to early identification of infectious disease pandemics and point to determinants of health that might otherwise have gone unnoticed [5].

## 2. Structure of this paper

This paper examines the ethical tensions that arise between the conflicting goals of advancing biomedical research and protecting patient privacy. We begin by examining the importance of privacy and the duty of medical confidentiality. We introduce the concept of selection bias to show how confidentiality and privacy can negatively affect the duty of beneficence. We then outline a particular version of the duty of beneficence—the duty of easy rescue—which applies only to situations in which it is possible to benefit others at no or minimal cost to oneself. We argue that the duty of easy rescue avoids some of the troublesome aspects of the more general duty of beneficence, and that it provides sufficient justification to override the duty to protect confidentiality and autonomy (as instantiated in the informed consent process) where this is necessary to avoid selection bias. Where the duty of easy rescue does not apply because there are significant risks involved in data sharing (where the ‘rescue’ is not ‘easy’), and where these risks cannot be minimized by security management, we argue that research can only ethically proceed without informed consent when obtaining consent would be impossible or impracticable, the public benefit of the research very significantly outweighs the risks, the public is adequately informed, and any resulting harms are compensated. On the basis of this discussion, we develop a framework that facilitates the ethical use of EHR data for the benefit of society.

This paper adds to the discussion of medical data sharing and confidentiality in several ways: firstly, we distinguish between the duties of easy rescue and general beneficence; secondly, we develop a framework that integrates these and other important considerations; and finally, we propose that public outreach and mechanisms of compensation are essential for the ethical facilitation of EHR data use.

## 3. Privacy and the duty of confidentiality

Health records can document sensitive information that patients might not desire others to know. Medical choices may reflect personal or religious values, such as decisions concerning reproductive medicine, organ donation or life support. Other information on health records may be seen as embarrassing or stigmatizing, including decisions concerning cosmetic surgery or psychiatric services. Finally, medical records may contain information, such as descriptions of psychiatric or substance misuse treatment, chronic debilitating illness, reproductive decisions or elective surgery, that could be used to discriminate against persons.

The right to privacy protects persons from unwanted intrusions into their personal life. Privacy is a complex concept that can take on different meanings in various contexts [8]. Privacy prevents information about individuals from being disseminated in inappropriate contexts. Medical information, for instance, is appropriately shared with professionals for the purposes of healthcare, but is protected by privacy in other spheres of life, such as employment [9]. Privacy protects against public surveillance and expectation, facilitating self-creation through trial and error free from judgement, which, in turn, is the basis for critical reflection on the types of societies and lives we want to lead [10]. In this work, we are concerned with a particular type of privacy, namely that which protects individuals from unwanted intrusions into their physical, mental and informational lives.

In the medical context, privacy is protected by medical confidentiality. Confidentiality in medicine dates to antiquity and it is included in the Hippocratic Oath: ‘Whatever, in connection with my professional practice, or not in connection with it, I see or hear, in the life of men, which ought not to be spoken of abroad, I will not divulge, as reckoning that all such should be kept secret’ (quoted in [11, p. 1934]). Exceptions to confidentiality are restricted to the cases of imminent danger, and even these are controversial [11–13]. Confidentiality is crucial for maintaining trust in the doctor–patient relationship. Without confidentiality, patients could not be sure that their sensitive information does not reach the wrong hands, and would therefore be less likely to share information [13]. Such concerns can lead to ‘privacy-protective’ behaviour, including self-medicating; avoidance of care; paying privately for procedures in order to avoid disclosure; and provision of false information, with negative consequences for quality of care. Roughly one in six patients report engaging in privacy-protective behaviour [14]. Conversely, trust improves therapeutic outcomes indirectly through increased satisfaction, adherence, disclosure and use of services [15].

Medical confidentiality precludes the sharing of identifiable medical information to third parties without a patient's autonomous and informed consent. Identifiable medical information is information, such as addresses or birth dates, that can be used to identify a particular individual. Identifiable information is different from anonymized patient information, which is information stripped of variables that can be used to directly identify individuals. Autonomy refers to an individual's freedom to live as they see fit without undue restraint from others, and is a fundamental value of liberal societies and many moral and political theories [16]. Respect for patient autonomy is recognized as one of the fundamental principles of biomedical ethics [17], and healthcare professionals must respect autonomy even when patients refuse life-saving treatment [18]. Respect for autonomy is important in its own right, but has also been shown to be medically beneficial [19].

Various international agreements, such as the World Medical Association's Declaration of Helsinki and International Code of Ethics, place duties on medical professionals to respect confidentiality and patients' rights to accept or refuse treatment [20,21]. In addition, national medical authorities and jurisdictions explicitly recognize confidentiality and respect for authority as duties of medical professionals [22–24].

Ordinarily, the primacy of respect for patient autonomy is uncontroversial—patients should, of course, have the right to decide whether and which procedures they wish to undergo. Yet it becomes more ethically problematic when it comes to control over information already stored which has implications for the health and well-being of others, especially when the risks involved in using that stored information are minimal. The next section demonstrates this problem in the context of selection bias and EHR research.

## 4. Informed consent and selection bias

Informed consent is a basic requirement for human subjects research [20]. The historical origins of this requirement are often traced to the Nuremberg Code and the original Declaration of Helsinki, which followed in the wake of medical atrocities conducted during the Second World War and the post-war years, though early versions of the requirement can be found in Prussian government directives as early as 1891 [25,26]. A common element in these studies was that harmful, sometimes lethal, experiments were performed on subjects against their will or without adequate understanding. The requirement of consent given with adequate understanding and without coercion protects against abuses.

At first glance, it seems that the problem of respecting autonomy and privacy while allowing qualified research access to health data can be solved by obtaining informed consent. Patients could be asked to contribute their data until a sufficiently large database is established. Unfortunately, there are a number of difficulties with this approach.

Firstly, informed consent may be difficult to achieve. Often, informed consent is equated with the signing of consent forms, with the assumption that patients understand the information conveyed in them. These forms can be long and complicated lists used to shield organizations from liability instead of truly informing people [17]. Because of the amount and complexity of information presented in such forms, some patients fail to read or understand them [27]. They may be presented when the patient is very sick, and patients may feel pressured into participation or not know that they can refuse [28].

Secondly, and very importantly, medical records exist whether they are used for research or not. Because of this, the option of declining to consent to research access to EHR data does not guarantee the safety of information stored in such records. EHR data are stored in clinics and hospitals, and they are, therefore, subject to risks of theft and misuse regardless of the use to which they are put. The US Department of Health and Human Services (DHHS) keeps a list of instances in which medical information relating to 500 or more patients has been lost or stolen (known as a privacy breach and notifiable by law). The majority of breaches occur in clinics and hospitals, and are often due to the theft of unencrypted computers or memory devices [29]. There are few instances of breaches occurring in research institutions. Indeed, access to EHR data can be structured such that medical data remain at their source [30]. Under such an arrangement, researchers would have remote access from a high-security environment, with no physical copies, adding little additional breach risk. Non-consenters would be subject to a degree of breach risk similar to that of those who do consent. Although the requirement of informed consent respects the autonomy of patients, it is important to note that it does little to protect the privacy of information stored in EHRs.

Finally, the requirement of informed consent significantly reduces the quality and amount of data available for research through selection bias [31,32]. Results obtained from research on sample datasets will not hold true if the samples are not representative of the population to which the research applies. For example, a medication may have different effects on old and young patients [33]. The effects of a drug on a sample of young persons might, therefore, not be a good guide to its effects on older persons. To circumvent this problem, researchers attempt to create samples that are an accurate representation of the general population so that their results can be of general use. This cannot be done if some do not consent, because those who do not consent are not included. Two systematic reviews have shown differences between consenters and non-consenters [31,32]. In one of these, researchers compared the age, sex, race, education, income and health status of persons who did and did not consent with observational research on their medical records across 17 studies [32]. They found that non-consenters differed from consenters on all six measures in an unpredictable way that could not be corrected for statistically. A more recent review supplemented these findings with 21 additional studies and three further outcome measures (mental health status, functioning and lifestyle factors) [31]. It found overwhelming evidence that consent and the type of consent do have an impact on the characteristics of the individuals who are included in clinical research studies, adding that ‘[it] is difficult to dispute this evidence’ [31].

Whether the magnitude of distortion introduced by selection bias is severe enough to warrant concern has recently been questioned [34]. In their article, Rothstein and Shoben [34] argue that the amount of bias created by consent requirements has been overstated, and is likely to be small rather than large; that this bias can be reduced by statistical techniques; and, finally, that residual effects of consent bias that remain after statistical control are below an acceptable level of imprecision. The authors base these conclusions on numerical scenarios presented as part of a description of a hypothetical study, in which the magnitude of bias is indeed small. However, the authors provide calculations for only a few of the many possible numerical scenarios. In a response to this article, Groenwold *et al*. [35] showed that a wide range of values for consent bias are possible in the hypothetical study used by Rothstein and Shoben, many of which are extremely high. Groenwold and colleagues point out that the true degree of bias cannot be known, because the exposure to the variable of interest in a particular study and outcomes of the population that decline consent remain unobserved. Thus, statistical adjustment for selection bias is at best only partially possible using circumstantial evidence [35]. Therefore, it cannot be said that the magnitude of bias introduced by consent requirements is always or generally below an acceptable level of imprecision; there are many instances in which the level of distortion is likely to be very high.

The problem of selection bias is especially acute for EHR research. Research performed on large datasets has high statistical power such that even small differences between groups may be statistically significant [31]. In addition, consent requirements drastically reduce sample size. In the recent review, approximately half of patients consented [31]. When a consent requirement was introduced for notification to the Hamburg Cancer Registry, registration fell by 70%—after which research on the Registry was discontinued [36]. Statistical simulations show that even small selection biases can have effects large enough to produce false results [35,37].

It is clear that the requirement of consent at times introduces significant hurdles for biomedical knowledge generation. Because biomedical knowledge translates into treatments that save lives, alleviate or eradicate disease, and improve well-being, longevity and health, informed consent requirements can impede the achievement of these highly important goals. Therefore, they require strong justification. In the cases of invasive and/or dangerous clinical research, consent is necessary to protect individuals from exploitation, deception, coercion and harm. But in the present context of research performed on pre-existing records, this justification does not obtain. In the next section, we argue that, where the risks involved in EHR data sharing are or can be reduced to minimal, there is a duty of *easy rescue* to share EHR data for responsible and beneficial biomedical research. We argue that the duty of easy rescue strongly motivates EHR data sharing independently of the effects of selection bias.

## 5. Duty of easy rescue

One minimal theory of moral obligation can be called a duty of easy rescue. Peter Singer famously described the following thought experiment:

If I am walking past a shallow pond and see a child drowning in it, I ought to wade in and pull the child out. This will mean getting my clothes muddy, but this is insignificant, while the death of the child would presumably be a very bad thing [38].

The thought experiment illustrates a situation in which a person can benefit another greatly at minimal cost. Such situations are intuitively different from situations in which benefitting others is associated with large costs or risks. A person who attempts to rescue a drowning child in deep waters with strong currents, for example, is endangering their own life for the benefit of another. Although we admire and praise such people, we would not necessarily find fault with others who failed to act in a similarly selfless way. However, this is not the case where the rescue is easy: leaving a child to drown in a shallow pool is morally abhorrent.

The duty of easy rescue can be formalized in this way:

*Duty of easy rescue*. When the cost to *X* of performing some action, *G*, is small, and the benefit to *Y* is large, then *X* ought to *G*.

This principle can take a collective form. Call this ‘collective duty of easy rescue’:

*Collective duty of easy recue*. When the benefit to *Y* is large of *G*-ing, and the cost to each of *X*_1_ … *X_n_* is small of each *G*-ing, then each of *X*_1_ … *X_n_* ought to *G*.

To take an example, if each person in the population could donate a few millilitres of blood easily (say some special vial was created that could be posted to each person), and collectively this would solve the blood supply shortage, then each person ought to donate a few millilitres of blood. Doing so would literally be life-saving, at no cost and minimal discomfort to the donors.

In the case of EHR research, the cost is that which occurs to individuals contributing data to EHR research. The collective benefits are to those who benefit in society, or perhaps even globally, from such research being conducted. For example, research into the side effects of statins will gather data from all people in a society on statins, and the benefits are to not only the very same population but also those in other societies who can learn from such research. This includes most Western societies in the case of statins.

## 6. Other moral duties and research

We have argued that there could be a duty of easy rescue to engage in health records research. This is a subset of a wider duty of beneficence, to help others. There are other appropriate ends of moral behaviour besides promoting well-being, such as respect for persons and justice [17]. The collective duty of easy rescue could be extended to a general theory of minimal collective moral obligation, though a complete description of this is beyond the scope of this article. For example, if a person can, at minimal cost to himself, contribute to a project that collectively relieves great injustice, that person should contribute to that project. We believe much health records research would not only improve people's lives but also reduce injustice. If so, similar arguments would apply.

Moral duties can conflict. Duties of beneficence can conflict with duties to relieve injustice, just as considerations of utility can conflict with equality [39]. How one balances conflicting moral considerations or duties is a complex general question, not particular to EHR research, and again is beyond the scope of this article. What we have argued is that there can be a *pro tanto* moral obligation to engage in health records research, and one such instantiation can be based on a duty of beneficence, in this case a collective duty of easy rescue. If such research increased injustice (say by further marginalizing a vulnerable or victimized population) that would be a moral reason not to engage in it which would have to be considered and balanced against considerations of beneficence in the particular context in which it emerges.

Unlike Singer's pond example, life is almost always uncertain. One cannot be sure that engaging in a piece of health records research will bring about benefits. But, this is true of signing an organ donor card—one cannot be sure that a compatible organ donor recipient will be available. As the probability that a beneficial outcome will result decreases, the cost that the duty of easy rescue requires of individuals reduces. However, we argue that the costs of most EHR research are very minimal.

## 7. Weighing the duties of confidentiality and easy rescue

Just as it would be contemptible to refuse to rescue a drowning child because one does not wish to get one's clothes wet, it would be contemptible to decline to contribute towards the eradication of disease and alleviation of suffering because of some similarly trivial cost. Indeed, in France, Austria and some other European countries, there are laws requiring easy rescue, such as offering resuscitation if one is passing by and able to assist. In EHR research, risk is the most important cost of data contribution, as data sharing itself is not burdensome. Benefits of data sharing accrue passively, with no active participation needed. Instead, the costs of data sharing amount to the additional risk of privacy breaches and consequent harm. In the following section, we examine whether and to what extent the risks associated with EHR research are minimal.

## 8. Minimal risk

Research that carries minimal risk is treated differently from research with greater than minimal risk in some regulations. For example, the DHHS allows waivers for informed consent requirements for minimally risky research where it would be impracticable to obtain consent, and certain other conditions are met [40]. The concept of minimal risk is important to our discussion because the duty of easy rescue applies to situations where great benefit to others can be obtained at minimal risk or burden to individuals.

There are a number of different definitions of minimal risk [41]. The DHHS defines as minimal risks those where ‘the probability and magnitude of harm or discomfort anticipated in the research are not greater in and of themselves than those ordinarily encountered in daily life or during the performance of routine physical or psychological examinations or tests' [40]. This definition encompasses two tests for minimal risk: where the probability and magnitude of harm are not greater than those (i) ordinarily encountered in daily life or (ii) during the performance of routine physical or psychological examinations or tests.

A paradigmatic risk of daily living is that involved in driving [41]. According to the US Census Bureau, more than 33 000 people died, and more than 2 million people were injured, in road traffic accidents in the USA in 2009 alone [42]. Given the 2014 population estimate of 318.9 million, this corresponds to a roughly 0.01% chance of death and 0.7% chance of injury per person per year. How does this compare with the risks of EHR research?

Estimating privacy harms is notoriously difficult [43]. However, we can begin to answer this question by looking at the DHHS's list of breaches affecting 500 or more persons. Adding up the individual breaches on the DHHS website, an estimated 803 600 EHRs were breached in 2010, which is reflective of the average of 1 067 114 such records breached annually from the end of December 2009 until the end of April 2016 [29]. The vast majority of these breaches stem from ‘business associates’: just three of these were responsible for 6 400 000 of the total 6 936 238 breaches. The businesses in question are data management and EHR providers: they are not the type of businesses that carry out health research. Importantly, these records would have been breached whether they were used for research purposes or not. Healthcare providers were responsible for only 68 735 annual breaches, or a 0.02% chance of record breach per person per year, given a population estimate of 318.9 million. Note that this is the number of breaches arising from healthcare providers in general. The number of breaches resulting from researchers is an unknown subset of this number. This figure should be seen as a very rough approximation, useful only to indicate the order of magnitude that real breach risks are likely to be near. Not every American has an EHR, and many of the breaches stemming from healthcare providers will be from primary care and not from research. The actual degree of risk is unknown.

Furthermore, a fair comparison requires an estimate of the number of persons who were harmed by these breaches, which is a subgroup of the total. Not all EHRs contain sensitive information, and only some breached records will be used for nefarious purposes. The actual number of instances in which sensitive information has been gleaned from privacy breaches resulting from research activities and then used for harmful purposes is unknown, but it is evidently smaller than the number of persons injured in road traffic accidents, even if we were to assume that all breaches result in harm equivalent to injuries arising from road traffic accidents.

EHR research also fares well on the second proposed test of minimal risk. EHRs are routinely used in primary care. The risks of EHR data breaches in primary care are the same as the risks of breaches in research, with the exceptions that primary care uses of EHRs are more frequent, and they may not be protected by the same level of security. The risks of EHR data sharing for research are not greater than the risks of EHR data sharing for the purposes of primary care. They qualify as minimal under both tests.

## 9. The charitable contribution standard

An important caveat is that circumventing consent requirements would impose a burden on persons regardless of their wishes. By contrast, contribution to research is often seen as ethically supererogatory: it is praiseworthy to contribute, but not blameworthy to refrain from doing so. Indeed, a third definition of minimal risk has been proposed that argues that risks stemming from research participation are minimal when they are no greater than acceptable risks arising from participation in charitable activities [44]. Can the charitable contribution standard be met by a policy that does not require informed consent for minimally risky EHR research?

We argue that it can. We often accept personal burdens for the benefit of society at large. Taxation and jury duty are broadscale examples of this [45]. In the medical context, we accept a number of risks and burdens in order to contribute to the advancement of medicine and health in general. For example, we are obligated by law to be quarantined in the case of dangerous infectious disease, in order to protect those whom we otherwise might have endangered. Some countries, including the USA, mandate vaccinations for children by law, effectively imposing a very small risk on individuals in order to achieve the greater benefit of eradicating disease. Vaccines pose risks of a mild to moderate adverse reaction in the range of 1 in every 100 to 100 000 cases, as well as lower risks of more serious adverse reactions [46]. Although our above estimate of an annual incidence of 0.02% (1 in 5000) of record breaches is very rough indeed—and bearing in mind that not every breach will lead to harm—it seems comparable with this level of risk.

## 10. Greater than minimal risk

Some types of EHR research may carry greater than minimal risk. There are few persons with very rare diseases, for example, and these might consequently be easier to identify from a dataset. Studies of particularly sensitive topics— such as mental health or reproductive interventions—have a greater chance of leading to harm in the event of a breach than studies of non-sensitive topics. In addition, some studies may require fully identifiable data, such as birth date, occupation and address. Compared with studies that can use aggregate or de-identified data—that is, data that have been stripped of identifying information—these studies have a greater chance of leading to harm.

Where there is greater than minimal risk, confidentiality and autonomy concerns become correspondingly weightier. There are many significant advances that could be achieved by imposing moderate and/or severe burdens on society without consent—including ethically monumental projects such as eliminating global poverty, hunger, climate change and deforestation. According to a recent United Nations estimate, it would cost $267 bn per year to eradicate world hunger in a sustainable fashion [47]. If all 1.2 bn people in the developed world were to pay an equal share, world hunger could be eliminated for $222.5 per person annually. Although this may sound like a fair price to pay for such a worthy outcome, there are a number of other important projects—such as eliminating poverty more generally, providing universal education and combating climate change—which, together, would add up to an unsustainably large annual number. This is a reflection of a more general problem, namely that beneficence seems to require us to do too much. According to utilitarians, we should continue to sacrifice until the costs to us are larger than the benefits to others. This requires extraordinary sacrifices, such as the donation of one of our two kidneys.

There will always be problems to solve and limited resources to do so. This is not an issue in cases where solving the problem is not burdensome, and is only attended by minimal risks—as we argue is the case with most EHR research—but it is a problem for more demanding ethical codes such as utilitarianism. It is also a problem for EHR research with greater than minimal risks, in that it imposes a sacrifice (in the shape of moderate or serious risks) for the benefit of others which would not be unreasonable to refuse in the light of the commonly accepted norms related to charitable giving and prosocial behaviour generally.

For these reasons, we believe that consent should be sought for EHR research with greater than minimal risk. But consent should not always be given overriding importance, especially when it is impossible or impracticable to obtain, for example in old registers where many patients are deceased or difficult to trace due to relocation. In the previous section, we argued that, in cases of minimal risk, consent may be bypassed if collective benefits are large enough. Here, we argue that, in cases of greater than minimal risk, consent is one important consideration among many. It might still be outweighed by overwhelming public benefits, but this requires stronger justification. This is akin to the situation with medical confidentiality more broadly. For example, information that raises suspicion of abuse can be shared without consent. Additionally, healthcare practitioners must notify public health authorities of the name, sex and address of persons suspected of carrying certain infectious diseases under the UK Public Health (Control of Diseases) Act 1984 and Public Health (Infectious Diseases) Regulations 1988. UK doctors are also required to release information that might help to identify a person suspected of being involved in a road traffic accident under the Road Traffic Act 1988. Equally, there should be mechanisms in place that circumvent the informed consent procedure where this is demonstrably necessary for some equally important outcome, for instance, where there is a sufficient degree of certainty that the study will lead directly to the prevention of serious harm.

The use of such mechanisms should be relatively rare. We suspect that the vast majority of proposed EHR research carries only minimal risks of harm. Cases where there is moderate or severe likelihood of harm, where consent is impracticable to obtain, and where there is overwhelming public benefit are not your average research study. When these criteria do obtain, cases should be given serious scrutiny. This leads naturally to the important questions of who should decide in which category of risk a research proposal lies, and how the categories should be defined.

## 11. An electronic health record research authority

Current regulations in the UK, USA and some other jurisdictions allow for expedited or no ethical review of retrospective research on data collected for other purposes where there is minimal risk to the individuals to whom the data refer [48,49]. However, these flexibilities are often not used in practice, due to concerns over liability and a general ‘culture of caution’ [50]. Allowing research access to minimally risky EHR data without consent would require little, if any, legal action.

We propose that the default position of research ethics committees should be to grant access to minimally risky uses of patient data without the need for consent or lengthy authorization mechanisms. The benefits of such an approach go beyond the benefits of EHR research generally, as delays stemming from ethical review can cost lives [51].

Where there is greater than minimal risk, cases need to be considered on their own merits. We propose that a national or state-level EHR research authority be established and invested with the power to grant research exemptions for the requirement of informed consent where this is necessary for projects with significant societal value. Alternatively, pre-existing research authorities, such as the UK's Health Research Authority or the NHS Research Ethics Committee, could be charged with this duty. Such an authority would improve the current situation in a number of ways.

The current regulatory system in the UK, for example, is marred by multiple overlapping statutes and regulations that are interpreted differently by numerous approval bodies, which, in turn, apply varying frameworks for decision-making [50]. A single, expert authority applying a uniform, transparent framework would reduce this variation. It would lighten the workload of local research ethics committees, allowing them to focus on projects with higher risk, such as interventional clinical research. Importantly, it would reduce redundancy of ethical review. Much EHR research will involve retrieving records from many different localities, involving multiple local research ethics committees. A single committee is necessary to both properly interpret complex laws and regulations and ensure efficient review [52]. Such an authority would operate at the national level and, as a government body, would be directly accountable to elected officials. An additional layer of accountability could be invested in an EHR research authority oversight body, charged with auditing and enforcing adherence to strict safety and privacy protection protocols. The proposed authority would be staffed by full-time employees with expertise in research and computer technology, who would be similarly accountable to the oversight body. The authority would be charged with protecting the privacy of data sources. Researchers would apply to the authority with requests for data access according to pre-defined protocols. The authority would then judge each application on its merits, deciding according to transparent criteria whether the value of the research justifies access to EHR data.

The authority could be located in or operate a safe haven, a physically secure facility with exceptionally high levels of data security. The aggregation of records into a centralized database would, of course, increase the risk of privacy breaches as they would be warehoused in a single location. Thus, any breaches that did occur would affect a much larger number of individuals at once. However, data warehousing is not the only possible model. Distributed systems link together many individual data hubs, such as clinics. Under one model, a researcher wishing to access records for research purposes would despatch a request for records of interest to all hubs in the distributed network [30]. These would be collated at the individual hubs and sent to a trusted, responsible intermediary who would then assemble the input from all hubs into a dataset fit for research purposes.

The Sentinel Initiative set up by the US Food and Drug Administration (FDA) is an example of a distributed system. The Sentinel Initiative is an attempt to create an electronic system for the monitoring of medical products available on the market [53]. A pilot initiative, the Mini-Sentinel, already has access to millions of health records. Mini-Sentinel uses a distributed database model in which data remain with the data sources. The Sentinel operations centre distributes queries to sources, using analysis programs created for that purpose. Data sources run these programs, returning summary counts and de-identified data whenever possible. The operations centre then aggregates responses and sends them to the FDA. In this way, the identifiability of information is kept to a minimum. In addition, all data sources are required to adhere to safety standards to protect privacy.

A distributed system reduces the risk for privacy breaches, as it obviates the need for central storage. Research uses would only add risk to the extent that transmissions between hubs, intermediaries and researchers is vulnerable to hacking and insofar as the delivered data are open to theft and misuse from the researchers' side. This situation is akin to the level of risk already inherent in primary care, and it would not result in significant additional breach risk. Distributed networks have the advantage of data custodianship remaining with data originators [30]. Data collected by one person may not be recorded in the same way as data collected by another. Sources could then report their data in a standardized format, according to templates or analysis programs despatched by the authority.

Such a system would increase responsible research access to EHR data while safeguarding patient privacy. The additional risk of privacy breaches would be minimal, as there would be no centralized database to hack and data would remain where they are. The only additional privacy breach risk would stem from employees of the authority, researchers who receive the data and the transmission of queries and data between sources, the authority and researchers. As evidenced by the list of breaches affecting 500 persons or more, these are not the usual victims or perpetrators of theft or hacking. Even if a breach were to occur, the information stolen would be limited to the narrow set of information strictly necessary for a specific research query, and would thus not have much commercial or political value.

A great benefit of such a system is the capacity to deploy strategies to minimize privacy risks. In the next section, we draw on the above discussion to develop a risk-adapted framework for the ethical facilitation of beneficial EHR research.

## 12. The framework

**Figure 1.**
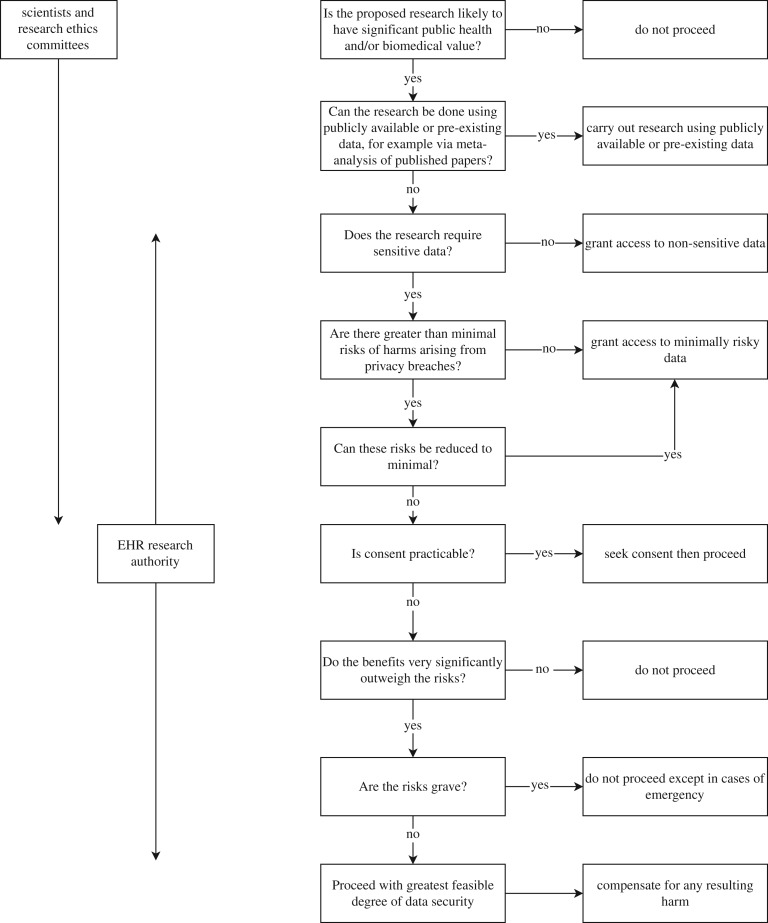
The proposed framework for ethical facilitation of EHR research. The flow chart in the middle and to the right of the figure represents key questions of beneficence and privacy protection. The boxes and arrows on the left indicate which oversight body is responsible for answering them. The overlap of oversight in the middle of the diagram indicates the possibility of appealing local review decisions to the proposed research authority.

How should research access to EHR data be managed? In this section, we propose a framework that fulfils the duties of beneficence as easy rescue while protecting privacy and autonomy. The framework is presented in figure 1. Each box in the flow chart represents a criterion that is important for determining whether and how proposed research on EHR data can ethically proceed. We look at each criterion in turn.

## 13. Is the proposed research likely to have significant public health and/or biomedical value?

Funding is limited and should be used on sound research that is likely to contribute to the goals of eradicating disease and improving well-being, or correcting injustice. There is a surprising amount of waste in biomedical research, owing to poor methodology, inappropriate selection of research topics, failure to review existing research, and incomplete or biased reporting [54]. Guidance on assessing methodological shortcomings and increasing value can be found, for example, in an excellent series of papers entitled ‘Increasing value: reducing waste’ published by *The Lancet* in 2014 and summarized in a recent review [55]. If the proposed research is not likely to have significant public health and/or biomedical value, the duties of beneficence and easy rescue cannot justify the use of EHR data. Such research should not proceed beyond institutional review.

## 14. Can the research be done using publicly available or pre-existing data?

Some research questions can be answered by combining pre-existing data. Additionally, some research questions can be answered by the use of publicly available datasets. The Personal Genome Project (http://www.personalgenomes.org/), for example, aims to make its genomic data publicly available for anyone to analyse. Research should be carried out using pre-existing resources whenever possible.

## 15. Does the research require sensitive data?

Not all medical data are sensitive. For example, the height of individuals or the number of routine check-ups they attend can be seen as non-sensitive. National statistical offices routinely collect demographic, economic, social and healthcare-related data for analysis and public policy purposes. Where the data in question do not relate to information that could be misused by third parties or cause harm to the data subjects in other ways, there is no reason to prevent access to it if so required for research projects with significant societal value. However, this should only be the case for information that is clearly not sensitive, such as eye colour. We consider as not sensitive any information which, by its nature, is unlikely to be used for unfair discrimination or exploitation.

## 16. Are there greater than minimal risks of harm arising from privacy breaches?

Setting exact thresholds for minimal risks is beyond the scope of this paper. However, the acceptable risks of routine medical, public health and everyday activities seem comparable with or greater than the risks of EHR research. This will not necessarily be the case for research on rare diseases and some vulnerable populations. Cases where risks of harm resulting from EHR research are clearly minimal should proceed without the need for informed consent. However, doubtful cases should be carefully assessed by research ethics committees. Contentious cases could be referred to the proposed research authority for adjudication. It is essential that both review boards and the proposed authority make use of transparent, easily accessible and clearly understandable guidelines for risk assessment.

## 17. Can these risks be reduced to minimal?

Where research proposals carry greater than minimal risks, researchers and research ethics committees should consider whether these can be reduced to minimal through data security measures. For example, a research proposal aimed at aggregating identifiable information pertaining to a particularly sensitive issue, such as suicide attempts, might be judged to carry greater than minimal risks to participants. This risk might be reduced to minimal through the use of a data safe haven, with researchers physically located inside a secure facility from which they cannot remove data. Some research projects can be carried out using de-identified data. Distributed database queries might be used to reduce the need for central aggregation.

## 18. Is consent practicable?

We argued above that consent should be sought for research projects that expose participants to greater than minimal risk. However, this is not always possible, as subjects may be deceased, have relocated, or simply fail to return phone calls or letters. Where consent is not practicable to obtain, and the benefits of proposed research very significantly outweigh the likely harm, researchers should be allowed to proceed without informed consent.

## 19. Are the risks grave?

EHR research should not proceed where there is significant possibility of grievous harm resulting from the research. Owing to the observational nature of EHR research, this category of risk is likely to be rare, but nonetheless provisions should exist to protect extraordinarily vulnerable patient groups. One example that might fall into this category would be proposed research on the psychological effects of extreme harassment, where the leaking of identifiable information could lead to victims' addresses being located by perpetrators of violence. Such research should only proceed in cases of overriding national or international emergencies.

## 20. Compensation

Our vision of the proposed research authority includes a mechanism of compensation for persons who have suffered harm as a result of privacy breaches, for two reasons. The first is a consideration of fairness. Although the benefits of EHR data research are likely to be spread across the entire patient population, the costs (in the form of privacy breaches) are borne by a few, unfortunate individuals through no fault of their own. One way to redress this unfairness is to award compensation to those who have been affected by privacy breaches. The costs of compensation could be spread across the entire population through taxation. In this way, the burdens of privacy breaches could be borne more equally and thus more fairly than they would be otherwise. The second reason is that a guarantee of fair and effective compensation for harms arising from privacy breaches is likely to contribute towards the acceptability of EHR research among the general public. As we argue in the next section, public support is crucial for a proposal that allows informed consent requirements to be circumvented.

Many jurisdictions already have some form of compensation for privacy breaches in place. Under the UK's Data Protection Act 1998, for example, ‘an individual who suffers damage by reason of any contravention by a data controller of any of the requirements of this Act is entitled to compensation from the data controller for that damage’ [56]. Thus, the data controller is responsible for compensation payments under UK legislation. There are pragmatic problems with ‘fault’ compensation systems such as this one: compensation claims must go through the courts, which means that many persons eligible for compensation fail to receive it, due to the practical hurdles in bringing a claim to court; in addition, claims that do proceed place large administrative costs and burdens on the legal system.

A number of countries offer ‘no-fault’ compensation to patients who have been injured due to medical errors and accidents [57]. A similar system could be set in place to help persons who have been exposed to harm as a result of their medical data being used for research. Persons who have been subjected to discrimination or exploitation could receive financial compensation from public funds according to the severity of harm. Instead of proving negligence or some other fault with data controllers, claimants would have to prove that harm resulted from a privacy breach. Where the harm resulted from unprofessional behaviour, the responsible researchers could face sanctions, including but not limited to revoking their access to EHR research data.

## 21. The importance of public outreach and trust

The importance of public outreach is demonstrated by the reaction to the UK government's recent care.data initiative. Medical data are routinely collected in the UK when patients visit hospitals. The care.data programme extends this data collection to all NHS-funded healthcare settings, including general practitioner (GP) visits, massively increasing the scope for medical data collection as GP visits are much more frequent than hospital visits. The programme faced serious opposition both from the public and from doctors, for a number of reasons, which resulted in the suspension of the programme. An in-depth analysis of the problems associated with care.data concluded that the mismanagement of public communication was one of the primary reasons for the programme's failure [58]. The public communications arm of care.data was limited to a leaflet entitled ‘Better information means better care’, which was supposed to reach 99% of UK households. However, a BBC poll found that less than a third of UK households had received the leaflet, which had been labelled ‘not fit for purpose’ by the Independent Information Governance Oversight Panel [58]. There was ‘no cohesive marketing campaign, no national TV campaign, no press conference, and the only supportive media was a video animation posted onto YouTube and the NHS England's website’ [58]. Carter and colleagues [59] suggest that care.data failed to secure public confidence due to ‘(i) defects in the warrants of trust provided for care.data, (ii) the implied rupture in the traditional role, expectations and duties of general practitioners, and (iii) uncertainty about the status of care.data as a public good’.

The case of care.data illustrates clearly the importance of public education, trust and outreach. It is crucial that the public is better informed of the value and limitations of observational research. A review of the literature on public perception of EHR data research reported that 11 of 13 included studies found a significant lack of understanding among the general public about the way their medical data are used [60]. The public shows a general distrust towards medical data sharing and wants to be asked for consent, but attitudes become more positive when the benefits and rationale of research are explained to them [60]. Public outreach and education explaining the benefits of well-designed EHR-based research performed under stringent privacy protection could go a long way towards maintaining trust in the healthcare system despite the removal of consent requirements.

## 22. Conclusion

We have argued that a duty of easy rescue applies to EHR data contribution because contributing data does not involve significant risks, costs or burdens; has large, potentially life-saving benefits; and refraining from doing so seriously hinders the provision of these benefits to individuals and groups. However, healthcare professionals also have a duty to respect patient privacy and confidentiality. In the case of EHR research, these duties conflict. This tension can be relieved either by offering research access only to de-identified data, data from persons who have consented, or by authorizing access to data without asking for consent in the first place.

Using de-identified data and data only from persons who have consented seriously undermines the quality of data available for research. Requiring consent will lead to distorted, and sometimes completely fallacious, results, which, in turn, lead to death and diseases that could have been easily avoided. These avoidable tragedies affect a very large number of persons.

Lifting the requirement of informed consent would cause a slight increase in the chance of privacy breaches above the level that would occur anyway. Of those persons who would not have consented to research access, a small subset will be affected by privacy breaches. Harm will occur only in a further subset. We argue that most EHR data research qualifies as minimally risky research, and should thus be exempted from informed consent requirements where this is necessary for research with significant public health and/or biomedical value. These risks are comparable to other risks in everyday life. Whether or not informed consent is deemed necessary, all feasible steps should be taken to reduce the risk of privacy breaches. This includes using data that are de-identified to the fullest extent compatible with research aims as well as the use of safe houses, distributed databases and best practice in data management.

In addition, we have argued that the establishment of an EHR research authority and oversight body utilizing a distributed database system with proper monitoring, security and ethical oversight minimizes the risk of privacy breaches without majorly impeding the research process. In cases of greater than minimal risk, consent should be sought when practicable, but it should be recognized as one of several important considerations and not accorded overriding status. We believe that such a system would greatly facilitate the ethical use of EHR-based research for the benefit of all.
